# Locus coeruleus neuromelanin, cognitive dysfunction, and brain metabolism in multiple system atrophy

**DOI:** 10.1007/s00415-025-12932-5

**Published:** 2025-02-11

**Authors:** Jacopo Pasquini, Hilmar P. Sigurdsson, Michael Firbank, Laura Best, Victoria Foster, Debra Galley, Ross Maxwell, Vincenzo Silani, Roberto Ceravolo, George Petrides, David J. Brooks, Nicola Pavese

**Affiliations:** 1 Clinical Ageing Research Unit, Translational and Clinical Research Institute, Faculty of Medical Sciences, Newcastle University, Campus for Ageing and Vitality, Westgate Road, Newcastle Upon Tyne, NE4 5PL UK; 2 Department of Clinical and Experimental Medicine, University of Pisa, Pisa, Italy; 3 Regional Neurosciences Centre, Royal Victoria Hospital, Belfast, UK; 4 Department of Neurology and Laboratory of Neuroscience, Istituto Auxologico Italiano IRCCS, Milan, Italy; 5 Department of Pathophysiology and Transplantation, Dino Ferrari Center, Università degli Studi di Milano, Milan, Italy; 6 Neurodegenerative Diseases Center, Azienda Ospedaliero Universitaria Pisana, Pisa, Italy; 7 Nuclear Medicine Department, Newcastle Upon Tyne Hospitals NHS Foundation Trust, Newcastle Upon Tyne, UK; 8 Department of Nuclear Medicine and PET Centre, Institute of Clinical Medicine, Aarhus University, 8200 Aarhus, Denmark

**Keywords:** Multiple system atrophy, Locus coeruleus, FDG-PET, Cognitive decline

## Abstract

**Background:**

Cognitive dysfunction is increasingly recognized in multiple system atrophy (MSA). Locus coeruleus (LC) integrity is associated with cognitive performance both in healthy controls (HC) and neurodegenerative conditions such as Parkinson’s disease (PD). Furthermore, cortical glucose hypometabolism is associated with impaired cognitive performance in MSA. However, knowledge about LC sub-regional degeneration and its association with cognitive dysfunction and cortical glucose metabolism is lacking.

**Objective:**

To investigate LC sub-regional involvement and its association with cognitive impairment and brain metabolism in MSA.

**Methods:**

Eleven MSA, eighteen PD, and eighteen HC participants were included in the study. Neuromelanin-sensitive MRI was used to determine rostral, middle and caudal LC neuromelanin signals. Brain glucose metabolism was investigated with [^18^F]Fluorodeoxyglucose PET (FDG-PET). The Montreal Cognitive Assessment (MoCA) was used as a measure of global cognition.

**Results:**

Middle LC neuromelanin signal was significantly reduced in MSA [*t*(43) = 3.70, corrected-*p* = 0.004] and PD [*t*(43) = 2.63, corrected-*p* = 0.041] compared to HC, while caudal LC was only reduced in MSA [*t*(43) = 2.82, corrected-*p* = 0.030]. In MSA, decreased rostral LC neuromelanin was associated with lower MoCA scores (*ρ* = 0.760, *p* = 0.006) which, in turn, were associated with lower frontal cortex glucose metabolism. An association between rostral LC neuromelanin signal and frontal cortex glucose metabolism was found in exploratory analyses.

**Conclusion:**

Loss of LC neuromelanin signal was found in MSA, the middle and caudal parts being targeted. Rostral LC neuromelanin signal loss was associated with both frontal cortex hypometabolism and lower MoCA scores. This pathophysiological link should be further investigated as the noradrenergic system transmission is amenable to pharmacological manipulation.

**Supplementary Information:**

The online version contains supplementary material available at 10.1007/s00415-025-12932-5.

## Introduction

Multiple system atrophy (MSA) is a neurodegenerative disease characterized by autonomic failure and a variable combination of ataxia, parkinsonism, and pyramidal signs [1]. Cognitive impairment is an increasingly recognized manifestation, although its prevalence and pathophysiology remain poorly understood [2]. Previous neuropathological studies have highlighted that people with MSA and cognitive impairment may have a greater burden of neuronal cytoplasmic inclusions in the hippocampus [3–5] and possibly increased amyloid beta and tau copathology [6–8].

Clinically, predictors of cognitive impairment in MSA are a longer disease duration, greater disability, and autonomic dysfunction, indicating an overall worse phenotype [9]. Previous neuroimaging studies have shown associations between cortical thinning in the anterior cortical regions [10, 11], subcortical atrophy [10, 12], and cognitive impairment in MSA. Reduced glucose metabolism in the prefrontal cortex was shown in MSA with cognitive impairment [13], and the presence of executive dysfunction was associated with prefrontal hypoperfusion [14] and hypometabolism [15].

In vivo locus coeruleus integrity, investigated with neuromelanin-sensitive MRI, is associated with better cognitive performances in older adults [16], while LC neuromelanin signal loss is associated with worse cognitive performance in Parkinson’s disease (PD) patients [17–20]. Previous studies investigating LC neuromelanin content in MSA showed similar or greater reductions compared to PD [21–24]. Sub-regional investigation of LC degeneration is now feasible and previous studies have shown differing patterns in PD and Alzheimer’s disease (AD): PD shows a greater involvement of the middle part and an overall caudal-to-rostral gradient of degeneration severity, while AD shows a greater involvement of the rostral part and an overall rostral-to-caudal gradient of degeneration severity [25, 26]. In MSA, LC sub-regional integrity and its association with cognitive performance has not been investigated. Therefore, the aims of this study were to investigate: (i) LC sub-regional integrity in MSA patients and compare it to that of PD and healthy controls (HCs); (ii) the associations between locus coeruleus integrity, cortical glucose metabolism measured with [^18^F]Fluorodeoxyglucose PET (FDG-PET), and global cognitive performance in people with MSA.

## Methods

### Participants

Fifty participants, aged between 45 to 80 years, were recruited for this study; thirteen with MSA, nineteen with PD, and eighteen healthy controls (HC) with no neurological abnormalities. One PD and two MSA participants were excluded from the analysis due to the presence of significant MRI motion artifacts and/or poor registration between FDG-PET and MRI images.

Participants were recruited between June 2019 and September 2021 from the Newcastle upon Tyne NHS Clinics for Research and Service in Themed Assessment (CRESTA), Campus for Ageing and Vitality, Newcastle upon Tyne, United Kingdom. Inclusion criteria were: age 45–80 years; for healthy controls (HC), absence of neurological symptoms or dysfunction, or MRI structural brain abnormalities; for MSA participants, a diagnosis according to Gilman et al. 2nd consensus criteria less than 3 years earlier [27]; for PD participants, a diagnosis according to the UK Brain Bank Criteria [28].

Seven MSA participants had predominant parkinsonism and four had a predominant cerebellar syndrome. All had a probable MSA diagnosis and no changes in diagnosis were made up to the time of writing.

Exclusion criteria for all participants included a diagnosis of other forms of atypical parkinsonism, significant cognitive impairment (Mini Mental State Examination [29] < 24 at screening visit) or meeting DSM V criteria for major neurocognitive disorder.

### Clinical assessments

All participants underwent clinical assessment on the day of their MRI and PET scans. Both MSA and PD participants were rated with the Montreal Cognitive Assessment (MoCA) [30], and the Scale for Outcomes in Parkinson’s Disease-autonomic dysfunction (SCOPA-AUT) [31]. The MoCA score was corrected for the educational level by adding one point if the participant was in education for 12 years or less, as indicated in the current version of the test. MSA participants were rated with the Unified MSA Rating Scale (UMSARS) [32]; PD participants were scored on the Movement Disorders Society-Unified Parkinson’s Disease Rating Scale (MDS-UPDRS) [33].

Antiparkinsonian medication was not withheld for participation in this study.

### MRI acquisition

All images were acquired with a 3T PET-MR System (Signa, GE Healthcare, Milwaukee, WI) equipped with a 32-channel head coil (Nova Medical, Wilmington, MA, USA). Foam padding was added between the participants’ head and the head coil.

A T1-weighted (T1w) 3D MRI sequence was acquired using a sagittal fast spoiled gradient recall (FSPGR) sequence with inversion time 400ms, echo time (TE) = 3 ms, repetition time (TR) = 7 ms, flip angle = 11° voxel size 1 × 1x1 mm and parallel acceleration factor = 2.

A neuromelanin-sensitive, fast spin-echo (FSE) T1-weighted sequence with TR = 600, TE = 12, flip angle 111°, two echo trains as described by Sasaki et al.[34] was also performed. Thirteen slices (2.5 mm + 0.3-mm gaps), perpendicular to the posterior aspect of fourth ventricle, were acquired with field of view 220 × 165 mm and matrix size 512 × 384. Three acquisitions were performed.

### FDG-PET acquisition

The MSA participants had FDG-PET on the same 3T PET-MR System used for MRI acquisition. Static FDG-PET was acquired in 3D list mode (matrix size—192 × 192, voxel size—1.56 × 1.56 ×2.78 mm) with decay, attenuation, scatter, and dead-time corrections. Each acquisition was performed 30 min after injection, over a 16-min period. Reconstruction of the 16-min acquisition was performed at the end of the acquisition as a series of short 2-min frames to allow inter-scan head motion correction. Standard PET reconstruction settings were used: time-of-flight mode with 8 iterations, 28 subsets and a 3-mm filter. Optimization of reconstruction parameters was additionally performed. A parametric image of FDG activity distribution using all coincident 511 keV emissions detected in this period was computed.

### Neuromelanin MRI data processing

To evaluate LC neuromelanin signal, the three FSE T1-weighted images of each participant were co-registered and averaged with SPM12 (Wellcome Trust Centre for Neuroimaging https://www.fil.ion.ucl.ac.uk/spm/) running on MATLAB (The MathWorks Inc., Natick, MA, USA). A slice-to-slice intensity variation correction was applied to scale each slice according to the mean intensity in a brain mask. This procedure allowed the correction for intensity inhomogeneities in contiguous slices without compromising LC neuromelanin-associated hyperintensity. LC neuromelanin content was calculated in the rostral, middle, and caudal regions following the method described in Doppler et al. [35]. A region of interest (ROI) was drawn on a MNI template following LC coordinates described in Keren et al. [36]. The ROIs was slightly enlarged to account for anatomical variation, generating a “search ROI” that included the hyperintense signal of the LC without including other equally-hyperintense structures. The search ROI was divided with two axial planes in three equal parts (rostral, middle, and caudal) to investigate sub-regional LC differences. A background ROI was placed in the central and median pons matching the extension of the LC search ROIs (Supplementary Fig. 1). This area contains the pontine nuclei and pontocerebellar crossing fibers, and no clearly hyperintense structures in a (FSE)T1-weighted image. Then each subject’s FSE image was co-registered with their anatomical T1 image using SPM12. The anatomical image was then spatially normalized using SPM’s segmentation tool. The LC “search ROIs” in MNI space were then transformed to each subject’s native FSE image using the inverse transformations. Then a custom MATLAB (MathWorks, Natick, MS, USA) script employing SPM functions was used to extract and calculate the mean intensity of the five brightest connected voxels from the “search ROIs” in each rostro-caudal subsection and from the background ROI.

The LC neuromelanin specific contrast was calculated as follows:

$$\text{LC NM contrast}=\frac{\text{mean ROI intensity}-\text{mean background ROI intensity}}{\text{mean background ROI intensity}}$$

Average (left and right) rostral, middle, and caudal LC neuromelanin contrast were calculated.

### FDG-PET data processing

FDG-PET and T1w data were analyzed using SPM12. First, using AFNI’s 3dresample function [37], all participants’ T1w images were re-oriented to have their origin lying on the AC-PC line, voxel coordinates in LPI reference space and resampled to an isotropic 1.5 mm resolution. The re-oriented and resampled images were then processed using the Mayo Clinic Adult Lifespan Template (https://www.nitrc.org/projects/mcalt/, [MCALT]) segmentation routine[38] that included (i) segmentation into tissue classes (GM, WM, CSF) using SPM12’s Unified Segmentation[39] and SPM tissue priors, (ii) bias field correction and (iii) normalization to MNI space. The total intracranial volume (TIV) was recorded (in ml). Finally, voxels with a greater probability of being CSF in the GM and WM images were removed.

Individual FDG-PET images were co-registered using rigid-body transformation to the participant’s segmented GM maps. For completeness, we corrected the co-registered images for partial volume effects (PVE) using the PETPVE12 toolbox [40] and the Müller-Gartner method with default settings. Due to slight inaccuracies in the correction of PET images using PVE, in the subsequent statistical analyses, non-PVE-corrected images were used. For completeness, we repeated the analysis with PVE-corrected images and obtained overlapping results. Standardized uptake value ratio (SUVR) images were then created by dividing voxel-wise FDG uptake with median uptake from the lateral occipital lobe (reference region) extracted from the Hammers atlas [41]. The occipital lobe was selected as a reference region in MSA because of the possible cerebellar involvement in this condition and the relative sparing of the occipital regions [42]. Finally, pre-processed FDG-PET images were smoothed with an 8-mm full-width at half maximum Gaussian kernel.

### Statistical analysis

Demographics and clinical characteristics are described as median and interquartile ranges (IQRs). Differences in age and sex across groups were tested through Kruskal–Wallis test and Chi-square test, respectively.

To investigate group differences in LC rostral, middle, and caudal regions (outcome variables), an omnibus “ANCOVA-type” analysis was set up for each region, with group (HC, MSA, and PD) as a factor and age as a covariate. Significant omnibus tests were followed up with post hoc comparisons (six one-tailed paired comparisons representing the different combinations of group differences, i.e., HC > MSA, HC < MSA, etc.) investigating differences between single subgroups. A family-wise error rate (FWER) correction was applied by dividing *p* values (indicated as p_FWER_ where performed) by the number of post hoc tests performed, in this case six for each group of post hoc tests. This analysis was carried out with permutation analysis for the linear model (PALM), a tool implemented in the FMRIB Software Library (FSL) designed to allow a “non-parametric” permutation inference for the general linear model [43]. *p* values were calculated with the PALM permutation algorithm (with 10,000 permutations). Levene’s test was used to ensure homogeneity of variances of the outcome variables across groups.

The associations between sub-regional LC neuromelanin, age, and MoCA scores in MSA and PD participants were tested with Spearman rank correlations. A voxel-wise analysis was carried out with SPM12 to investigate the association between whole-brain FDG uptake, MoCA scores, and sub-regional LC neuromelanin signal values. Results are reported following an initial voxel-wise thresholding at *p* < 0.001 uncorrected, followed by family-wise error (FWE) cluster correction. An exploratory cluster-forming threshold of *p* < 0.01 was also used to investigate the association between LC neuromelanin signal and FDG metabolism. The significance threshold for all statistical analyses was *p* < 0.05.

### Data availability

Data used in the preparation of this manuscript are available upon reasonable request.

## Results

No significant differences in age (*H* = 0.682, *p* = 0.711) or sex (*χ*^2^ = 1.848, *p* = 0.397) were present across the groups. Compared to the PD group, the MSA group had lower disease duration since diagnosis (*U* = 12, *p* < 0.001) and time since the reported onset of symptoms (*U* = 38, *p* = 0.005). Demographics and clinical characteristics are shown in Table 1.

**Table 1:** Demographics and clinical characteristics of multiple system atrophy (MSA), Parkinson’s disease (PD), and healthy controls (HC)

	Healthy controls	Multiple system atrophy	Parkinson’s disease	Statistical test, *p* value
Number, *n*	18	11 (7 MSA-P, 4 MSA-C)	18	–
Sex, M/F	7/11	6/5	11/7	*χ*^2^ = 1.848, * p* = 0.397
Age at enrollment, years (median, IQR)	58.5 (20.0)	64.0 (18.0)	64.0 (14.0)	*H* = 0.682, * p* = 0.711
Age at onset of symptoms (median, IQR)	–	59.9 (8.5)	55.0 (15.0)	*U* = 59, * p* = 0.076
Time since diagnosis, months (median, IQR)	–	9.1 (11)	59.1 (68)	*U* = 12, * p* < 0.001
Symptoms duration, months (median, IQR)	–	36.1 (18)	75.8 (62)	*U* = 38, * p* = 0.005
MoCA (median, IQR)	–	25 (3)	27.5 (4)	–
MDS-UPDRS I (median, IQR)	–	15 (12)	10 (8)	*U* = 52, * p* = 0.035
MDS-UPDRS II (median, IQR)	–	20 (8)	7.5 (9)	*U* = 17, * p* < 0.001
MDS-UPDRS III (median, IQR)	–	–	33 (23)	–
UMSARS I (median, IQR)	–	18 (7)	–	–
UMSARS II (median, IQR)	–	27 (12)	–	–
UMSARS IV (median, IQR)		3 (2)	–	–
SCOPA-AUT (median, IQR)	–	25 (16)	11.5 (13)	*U* = 30.5, * p* = 0.001

Uncorrected *p* values are shown

*IQR* interquartile range, *MDS-UPDRS* Movement Disorders Society-Unified Parkinson’s Disease Rating Scale, *MoCA* Montreal Cognitive Assessment, *MSA-P* multiple system atrophy-Parkinsonian type, *MSA-C* multiple system atrophy-cerebellar type, *SCOPA-AUT* Scales for Outcomes of Parkinson’s Disease-autonomic, *U* Mann–Whitney *U* test, *UMSARS* Unified Multiple System Atrophy Rating Scale

### Locus coeruleus neuromelanin signal in MSA and PD

Between-group significant differences in neuromelanin signal were found in the middle and caudal parts of the LC [middle: *F*(1,43) = 7.51, *p* = 0.001; caudal: *F*(1,43) = 4.48, *p* = 0.016]. No significant differences were found across groups in the rostral part. In the middle part, neuromelanin signal was reduced in both MSA and PD groups compared to controls [MSA: *t*(43) = 3.70, *p*_FWER_ = 0.004; PD: *t*(43) = 2.63, *p*_FWER_ = 0.041]. In the caudal part, only the MSA group showed a significant reduction compared to controls [*t*(43) = 2.82, *p*_FWER_ = 0.030], while the reduction in the PD group was not significant after multiple comparisons correction (*t*(43) = 2.12, *p*_FWER_ = 0.118).

**Fig. 1:**
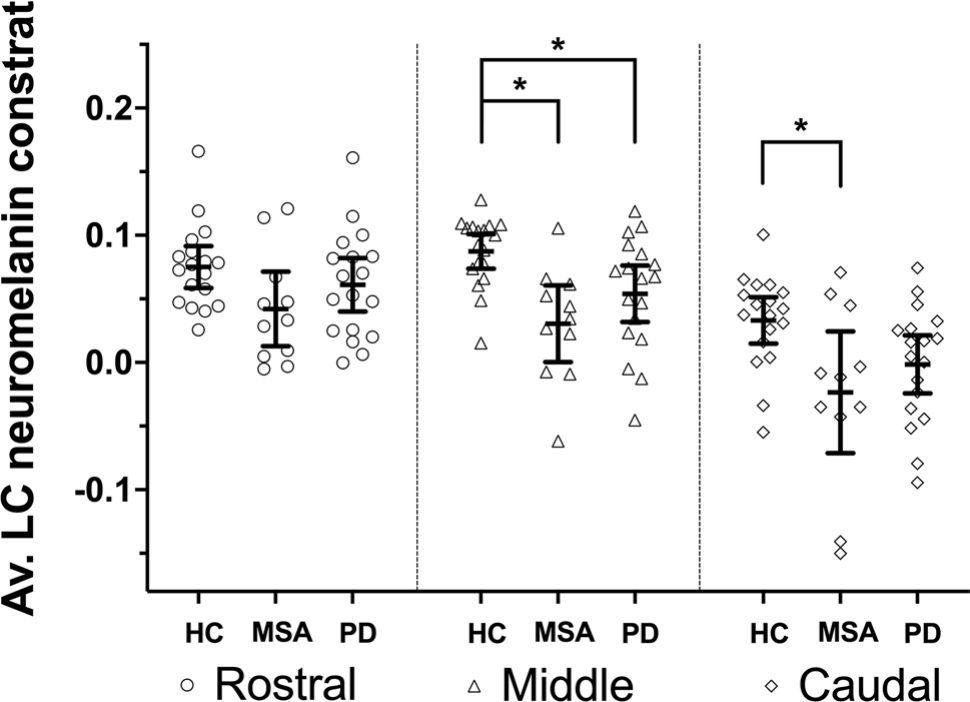
Line plots showing mean and 95% confidence intervals for locus coeruleus (LC) rostral, middle and caudal average (Av.) neuromelanin contrast in healthy controls (HC), multiple system atrophy (MSA) and Parkinson’s disease (PD). Individual values are also shown. Asterisks (*) indicate significant differences (after family-wise error rate correction) between single subgroups in post hoc tests that followed significant omnibus ANCOVA models

Figure 1 shows rostral, middle, and caudal LC neuromelanin content values in MSA, PD, and HC.

### Relationship between LC neuromelanin content, cognitive screening scores, and brain metabolism

In MSA, rostral LC neuromelanin content showed a significant direct association with MoCA scores [*ρ*(9) = 0.770, *p* = 0.006]. Middle and caudal LC neuromelanin signal were not significantly associated with MoCA scores [middle: *ρ*(9) = 0.484, *p* = 0.132; caudal: *ρ*(9) = 0.452; *p* = 0.163]. Age was not associated with MoCa scores or LC neuromelanin signal in MSA (all *p* values > 0.300).

In PD, no significant associations were found between MoCA scores, LC neuromelanin, and age; middle LC neuromelanin signal showed a non-significant association with MoCA scores [*ρ*(16) = 0.349, *p* = 0.155]. MoCA scores were not associated with age or LC neuromelanin signal in the PD group.

In the entire patient group (participants with MSA and PD), rostral and middle LC neuromelanin signal showed a direct association with MoCA scores [rostral: *ρ*(27) = 0.4906, *p* = 0.0069; middle: *ρ*(27) = 0.4913, *p* = 0.0068; Supplementary Fig. 2]. No association with caudal LC neuromelanin was found.

**Fig. 2:**
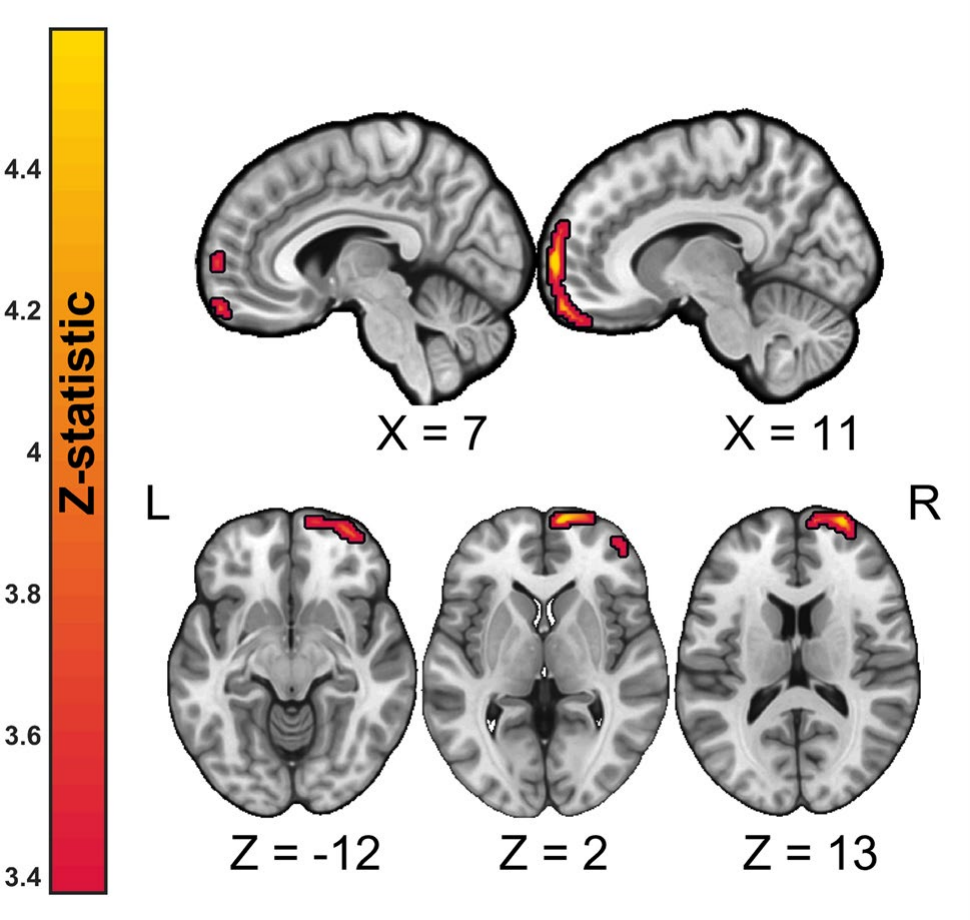
Anatomical localization of significant (voxel-wise thresholding at *p* < 0.001 uncorrected followed by family-wise error FWE cluster correction) direct associations between brain metabolism as measured by FDG-PET and Montreal Cognitive Assessment (MoCA) scores projected onto sections of a standard template in MNI space. The exact anatomical coordinates are referenced in the text and in Table 2. Top panel: sagittal view, lower panel: axial view. Numbers indicate MNI coordinates. *R* right, *L* left

**Fig. 3:**
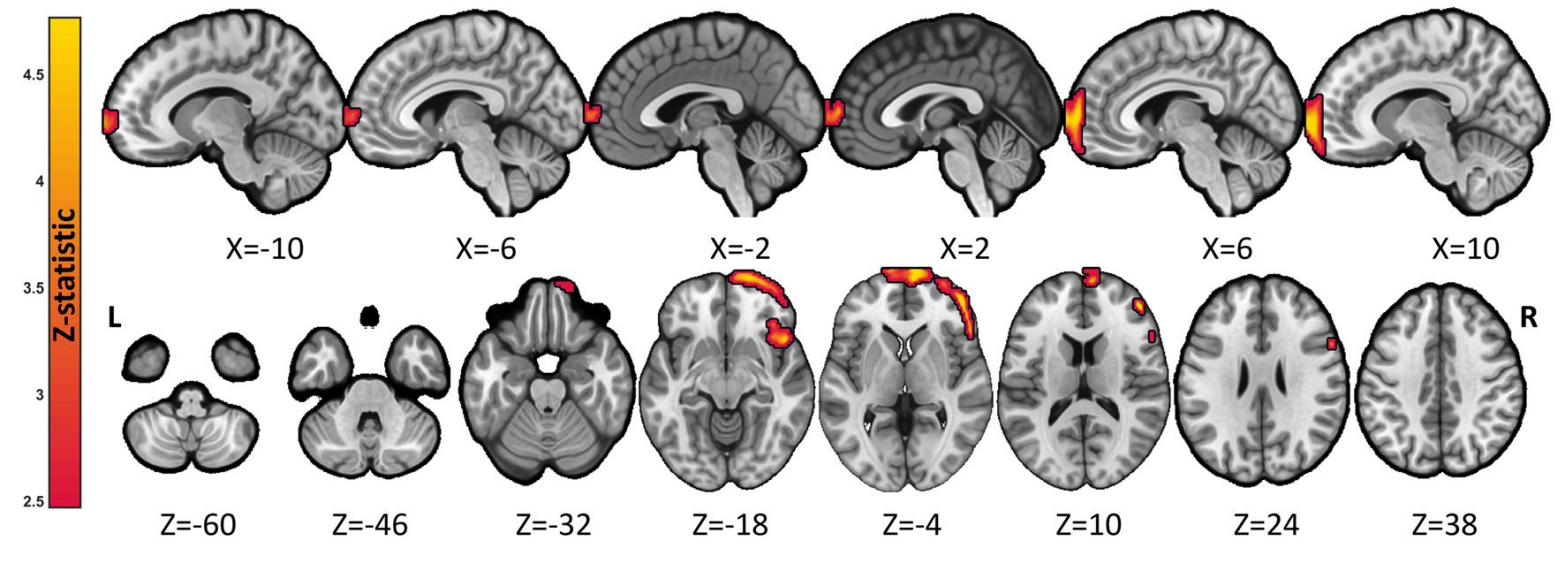
Anatomical localization of significant (exploratory voxel-wise thresholding at *p* < 0.01 uncorrected, followed by family-wise error FWE cluster correction) direct associations between brain metabolism as measured by FDG-PET and rostral locus coeruleus neuromelanin contrast scores projected onto sections of a standard template in MNI space. The exact anatomical coordinates are referenced in the Table 3. Top panel: sagittal view, middle panel: axial view, bottom panel: coronal view. Numbers indicate MNI coordinates

The voxel-wise analysis performed on FDG-PET images acquired in MSA participants showed a significant direct association between brain metabolism in the right medial superior frontal cortex [MNI: *X* 10, *Y* 64, *Z* 4, *t*(9) = 7.24, cluster size = 615] and MoCA scores (Fig. 2**, **Table 2). Furthermore, at the exploratory cluster-forming threshold of *p* < 0.01, rostral LC neuromelanin signal showed a significant direct association with FDG-PET metabolism in a cluster with peak coordinates located in the right middle frontal cortex (Fig. 3, Table 3).

**Table 2:** Significant clusters of associations between MoCA scores and brain FDG-PET metabolism at cluster-forming threshold *p* < 0.001 and p-FWE < 0.05 cluster-corrected

Cluster	Label	Side	#Voxels	*Z*-stat	MNI coordinates		
					*X*	*Y*	*Z*
1	Superior frontal gyrus, medial	R	615	4.1	10	64	4
	Superior frontal gyrus, dorsolateral	R			26	64	10
	Medial orbital gyrus	R			12	60	− 18

*R* right hemisphere, *L* left hemisphere, *MNI* Montreal Neurological Institute

**Table 3:** Significant clusters of associations between rostral LC neuromelanin signal and brain FDG-PET metabolism at cluster-forming threshold *p* < 0.01 and p-FWE < 0.05 cluster-corrected

Cluster	Label	Side	#Voxels	*Z*-stat	MNI coordinates		
					*X*	*Y*	*Z*
1	Middle frontal gyrus	R	1929	4.2	44	44	16
	Inferior frontal gyrus, opercular part	R			54	16	20
	Middle frontal gyrus	R			44	50	4

*R* right hemisphere, *L* left hemisphere, *MNI* Montreal Neurological Institute

## Discussion

In this study, LC sub-regional neuromelanin involvement and its association with cognitive scores and brain metabolism was investigated in MSA. A similar pattern of LC degeneration was found in MSA and PD groups compared to controls, with greatest involvement of the LC middle part. The MSA group also showed a significant reduction in the caudal part compared to controls. Furthermore, significant associations between reduced rostral LC neuromelanin signal, worse cognitive scores, and reduced brain FDG-PET uptake in frontal areas were found in MSA.

In PD, reduced neuromelanin signal in the LC middle part has previously been shown in a few studies [35, 44]. In our study, a significant neuromelanin signal reduction in middle LC was also shown in MSA. A small yet significant reduction was also observed in the LC caudal part in MSA compared to controls. Since previous studies investigating sub-regional LC degeneration in MSA are lacking, this finding can only be compared to previous investigations where the overall rostro-caudal LC neuromelanin signal was evaluated. One small study in nine MSA participants identified a greater reduction in LC neuromelanin signal in MSA compared to PD [21]; however, it should be acknowledged that mean disease duration (intended as time since diagnosis) in the MSA group was 4.7 years, while in this study, it was about 1 year. [21]. Another study from the same group involving 19 MSA-P and 11 MSA-C participants did not identify differences compared to PD [22]. A recent study with 14 MSA-P and 5 MSA-C participants showed overall reduced LC neuromelanin signal in MSA compared to PD without RBD but similar to PD with RBD [24]. The reductions we observed in the middle and caudal parts and the overlapping topographical degeneration pattern with PD extend those findings and are in keeping with existing neuropathological studies. Indeed, in MSA, a few studies reported LC macroscopic pallor [45], the presence of α-synuclein inclusions inside glial cells [46] and noradrenergic neurons [5], and overall severe neuronal loss compared to controls [46–48]. However, systematic neuropathological investigations with sub-regional characterization of LC neuromelanin in MSA are lacking. Future pathological studies providing more detailed analyses of the LC sub-regions would help the interpretation of these in vivo findings. Overall, the finding of a similar pattern of LC sub-regional degeneration in MSA compared to PD testifies to the vulnerability of these neuronal group to neurodegenerative processes involving alpha-synuclein pathology. This finding, coupled with a significantly shorter disease and symptoms duration in the MSA compared to the PD group, suggests a detectable, severe involvement of the LC in MSA since the early stages.

In MSA, reduced rostral LC neuromelanin signal was associated with reduced global cognitive functioning. MoCA scores were, in turn, associated with brain hypometabolism in a cluster involving the right medial and superior frontal cortices. In the exploratory analysis (cluster-forming threshold of *p* < 0.01), reduced LC neuromelanin signal was also associated with reduced brain metabolism in a cluster involving the right medial and inferior frontal cortices. These associations in partially overlapping clusters reinforce the role of LC connections to the cerebral cortex in mediating cognitive performances, especially attention and executive functions. To the best of the authors’ knowledge, the association between LC neuromelanin signal and cortical FDG-PET metabolism has been investigated in only one recent study in people with AD [49]. In that study, reduced LC neuromelanin signal was associated with reduced glucose metabolism in several clusters in the left frontoparietal cortices. Similar studies in parkinsonian disorders are currently lacking, despite growing evidence of the association between LC neuromelanin signal loss and cognitive dysfunction. The LC is the main source of central noradrenergic innervation and its terminals densely populate the cerebral cortex [50]. Biologically, noradrenaline modulates neuronal and synaptic homeostasis, glial activation, and neurovascular regulation [51–53]. Therefore, noradrenergic dysfunction could be a driver of reduced cortical glucose uptake and metabolism [54]. Future studies in larger MSA and PD cohorts should investigate the relationship between LC neuromelanin loss, cortical glucose metabolism and cognitive dysfunction in more detail. If a consequential effect of LC degeneration on reduced cortical glucose metabolism and cognitive decline is confirmed, the rationale for pharmacologically targeting the noradrenergic system with therapies would be strengthened. Nonetheless, in MSA, other factors are likely to play a role in cognitive decline, such as cholinergic loss, basal ganglia dysfunction, and white matter degeneration, and should not be overlooked. The interplay between the noradrenergic, dopaminergic, and cholinergic systems would be a further avenue to explore when investigating cognitive decline.

LC neuromelanin signal loss has been associated with worse cognitive performances both in healthy aging [16, 55, 56] and pathology including AD [26] and PD [17–19]. Specifically, higher rostral LC neuromelanin signal is associated with better memory performance in healthy older adults [56]. Therefore, even in the absence of a significant reduction in rostral LC signal in MSA, the relationship between rostral LC neuromelanin signal and MoCA scores extends those findings and emphasizes the pathophysiological role of LC integrity in cognitive functioning. From a metabolic standpoint, the association between cognitive scores and FDG-PET metabolism in prefrontal areas has previously been shown. In 84 MSA patients, brain hypometabolism in the left middle and superior frontal lobe was identified in participants with dementia or mild cognitive impairment compared to those with preserved cognition [13]. Furthermore, prefrontal hypometabolism [15, 57] and hypoperfusion [14] have been associated with impaired performances in attention, executive, and language domains.

Several limitations of this study should be addressed. The sample sizes of the individual groups were relatively small and the analysis may have missed less pronounced between-group differences and associations due to low statistical power. The MSA participants included were either predominantly parkinsonian or cerebellar, but sample size prevented further subgroup analysis. However, some studies suggested that cognitive impairment may differ in the parkinsonian and cerebellar subtypes [58, 59] due to their different pathological involvement and the presence or absence of copathologies [6]. Future studies in vivo should try to characterize differences and pathophysiological mechanisms between the two subtypes. Only the MSA participants had FDG-PET; therefore, the associations between LC neuromelanin signal, cognitive scores, and cortical metabolism could only be performed in this cohort. Cognitive functioning was rated with the MoCA, which is a global cognitive screening test; future studies with formal cognitive testing may elucidate further associations between LC neuromelanin loss and specific cognitive functions. Unlike previous studies [17, 19], in PD, we did not identify a relationship between LC degeneration and cognitive scores. However, it should be noted that the PD group was smaller compared to those studies and no formal neuropsychological battery testing was performed; therefore, our study was likely not powered to identify relationships with cognitive domains. The locus coeruleus noradrenergic activity influences several physiological processes beyond cognition, such as, e.g., cardiorespiratory responses. Because of sample size limitations, we did not explore such associations, which should be investigated in future studies.

In conclusion, in MSA, we identified a pattern of LC degeneration that resembles that of PD, though to a greater degree. Reduced rostral LC neuromelanin signal loss was associated with overall worse cognitive functioning in MSA and with decreased glucose metabolism in cortical frontal areas. Furthermore, the exploratory analysis also showed associations between reduced rostral LC neuromelanin signal and reduced frontal cortex glucose metabolism. These findings emphasize the vulnerability of the LC neurons in neurodegeneration with Lewy body pathology and the pathophysiological link to cognitive impairment. The relationship between LC neuromelanin signal loss and decreased cortical frontal glucose metabolism, which has also been described in AD, could point to a pathophysiological mechanism of the LC in maintaining executive functions in health and disease.

## Supplementary Information

Below is the link to the electronic supplementary material.

Supplementary file1 (TIF 621 KB)

Supplementary file2 (TIF 359 KB)
