# Supplementary figures and images for: Locus coeruleus neuromelanin, cognitive dysfunction, and brain metabolism in multiple system atrophy
Source: J Neurol. 2025 Feb 11;272(3):195. doi: 10.1007/s00415-025-12932-5 (PMC11814031; doi:10.1007/s00415-025-12932-5)

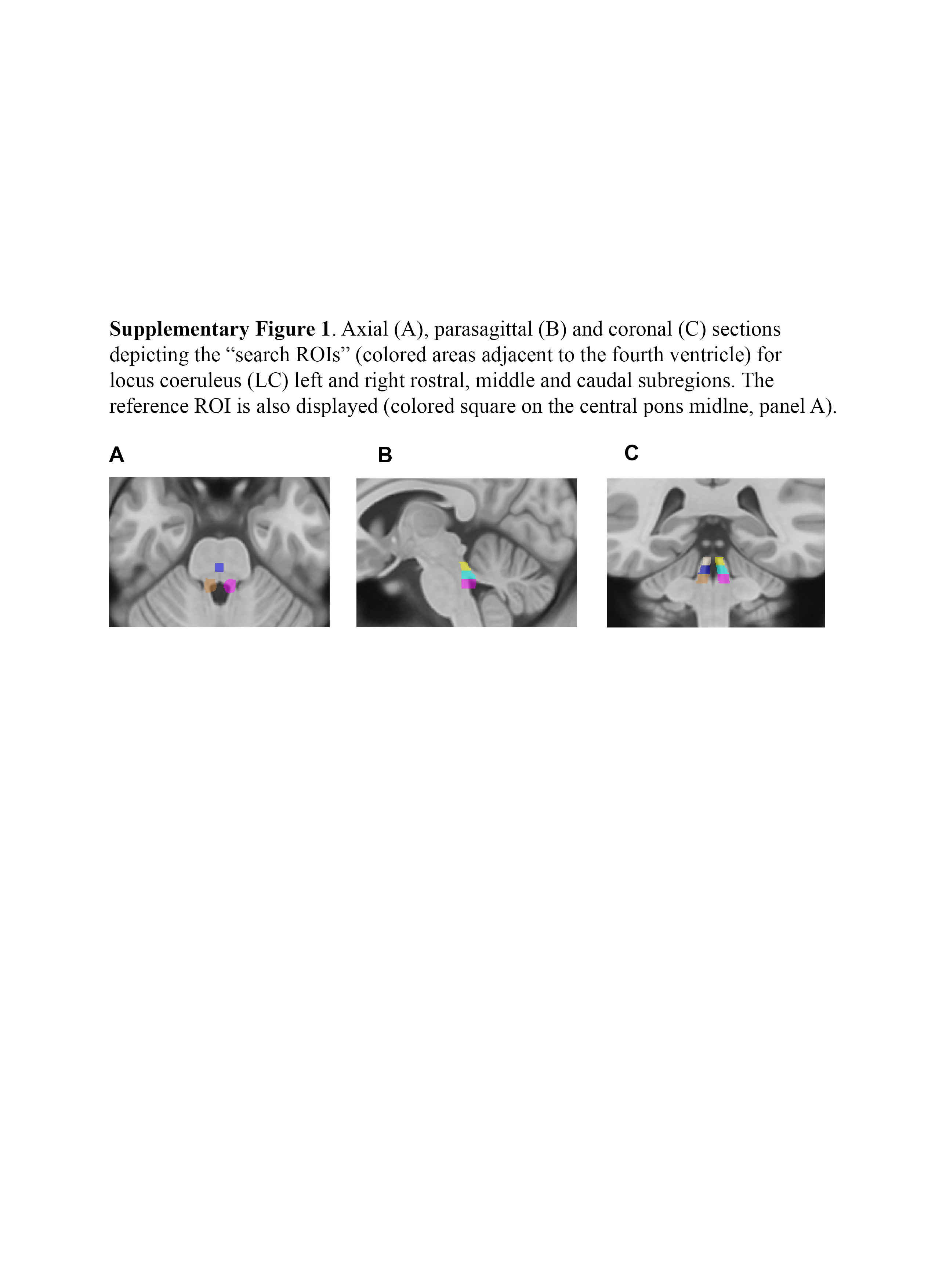

Supplement: Supplementary file 1 — Supplementary file1 (TIF 621 KB) [file 415_2025_12932_MOESM1_ESM.tif]

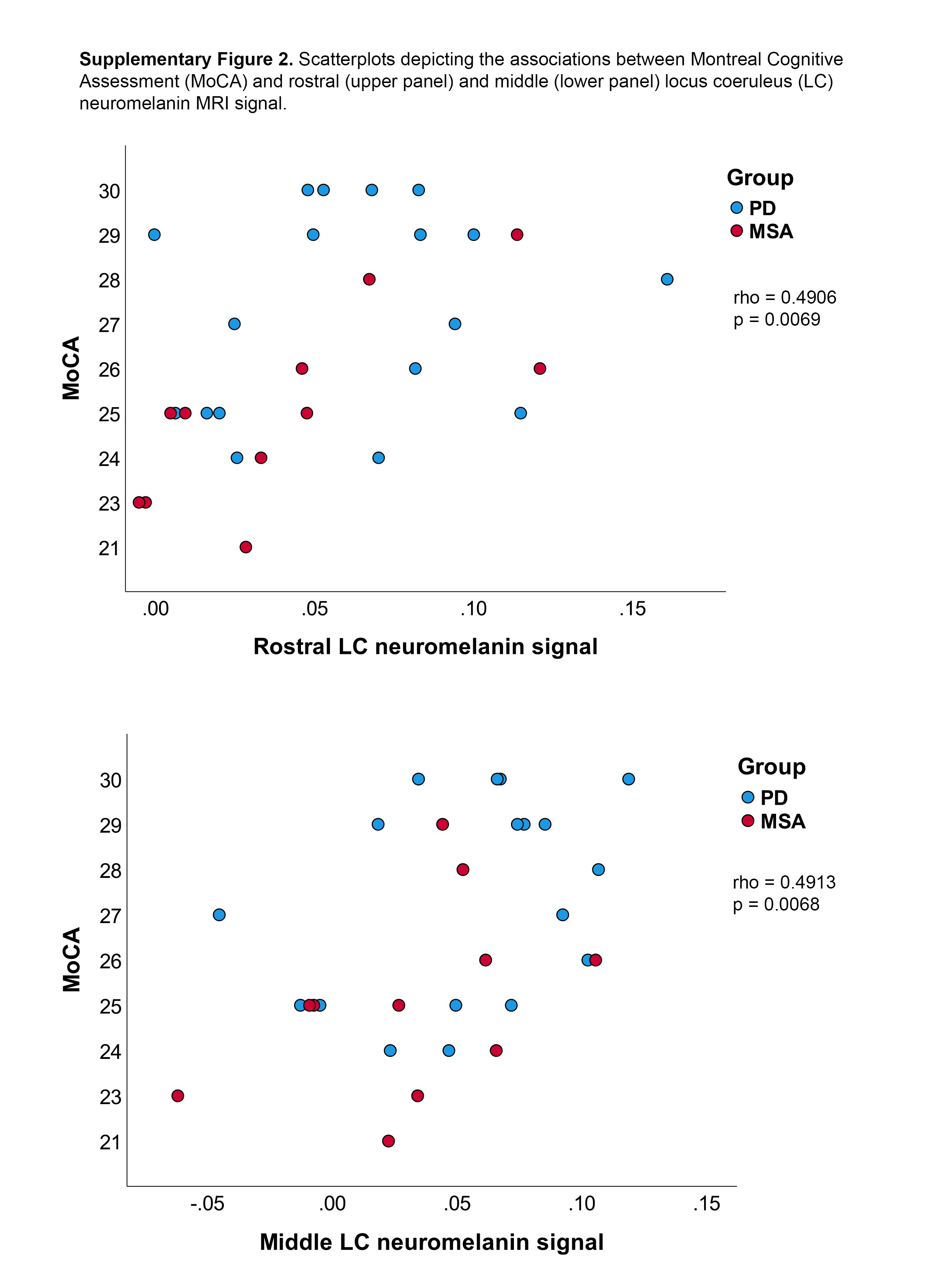

Supplement: Supplementary file 2 — Supplementary file2 (TIF 359 KB) [file 415_2025_12932_MOESM2_ESM.tif]
